# Gender- and age-related differences in homocysteine concentration: a cross-sectional study of the general population of China

**DOI:** 10.1038/s41598-020-74596-7

**Published:** 2020-10-15

**Authors:** Ranran Xu, Fei Huang, Yiru Wang, Qingquan Liu, Yongman Lv, Qian Zhang

**Affiliations:** 1 Department of Nephrology, Tongji Hospital, Tongji Medical College, Huazhong University of Science and Technology, 1095 Jie Fang Avenue, Hankou, Wuhan, 430030 People’s Republic of China; 2 Department of Endocrinology, Tongji Hospital, Tongji Medical College, Huazhong University of Science and Technology, 1095 Jie Fang Avenue, Hankou, Wuhan, 430030 People’s Republic of China; 3 Health Management Centre, Tongji Hospital, Tongji Medical College, Huazhong University of Science and Technology, 1095 Jie Fang Avenue, Hankou, Wuhan, 430030 People’s Republic of China

**Keywords:** Biomarkers, Cardiology, Medical research, Risk factors

## Abstract

The primary goals of this study were to evaluate the gender- and age-related differences in homocysteine concentration in the general population of China and possible influencing factors. A total of 7872 subjects, divided into male and female groups, participated in this retrospective study. The average homocysteine level, prevalence of hyperhomocysteinemia, and independent factors affecting homocysteine concentration were analyzed. The homocysteine level was significantly higher in males than in females in each age range (aged 20–30, aged 30–40, aged 40–50, aged 50–60, aged 60–80, aged over 80) (*P* < 0.0001), and the trend did not abate with age. The homocysteine concentration first decreased and then increased, being lowest at 30–50 years of age and significantly increased after 50 years of age. Factors associated with homocysteine concentration in males were smoking status (current smokers versus ex-smokers: β: 0.112), estimated glomerular filtration rate (*β* =  − 0.192), blood urea nitrogen (*β* =  − 0.14), diastolic blood pressure (*β* =  − 0.113), free triiodothyronine (*β* =  − 0.091), serum potassium (*β* =  − 0.107) and cystatin C (*β* = 0.173). In females, independent factors associated with homocysteine concentration were cystatin C (*β* = 0.319), albumin (*β* = 0.227), free thyroxine (*β* = 0.179), age (*β* = 0.148), free triiodothyronine (*β* =  − 0.217) and serum potassium (*β* =  − 0.153). The homocysteine level was significantly higher in males than in females and increased markedly after 50 years of age in both groups. The independent factors associated with increased homocysteine concentration differed between males and females.

## Introduction

Homocysteine (Hcy), which has caught the attention of researchers and clinicians in recent years, is a recognized biomarker and an established independent risk factor for various diseases^1^, including cardiovascular disease (CVD)^2–6^, neurocognitive disorders^7,8^ and osteoporotic fracture^9,10^.

Although interest in Hcy and hyperhomocysteinemia is growing, few studies have assessed the difference in Hcy concentration in normal subjects by age and gender. Knowing the level of Hcy in a healthy population and its fluctuations throughout life and differences between genders is crucial for clinicians’ correct interpretation of laboratory test results, especially when using the biomarkers in disease diagnosis, prognosis, or surveillance. Hcy reference intervals, stratified by age and gender, have been investigated in some Western laboratories^11–14^, but so far this has not been done in China, although several reports have shown racial and ethnic differences in plasma Hcy levels^15,16^. In China, clinicians refer to Western white Hcy levels in daily practice. As it has been established that there are racial and ethnic differences in plasma Hcy levels, it is necessary to investigate the Hcy level of Chinese people to establish a regional reference interval applicable to Chinese people.

There is a considerable gap in our understanding of Hcy, not only in the trends of Hcy concentrations in populations, but also in how gender affects the Hcy concentration in the blood and the physiological processes affected by Hcy. Hcy is metabolized mainly through the remethylation pathway and the transulfuration pathway. Severe hyperhomocysteinemia is usually due to rare genetic defects resulting in deficiencies of enzymes involved in Hcy metabolism. Mild hyperhomocysteinemia seen in fasting is due to mild impairment in the methylation pathway (i.e. folate or vitamin B12 deficiency)^17^. It has been reported that vitamin B12 or folate^8^ deficiency was the cause in two thirds of patients with hyperhomocysteinemia studied. The vitamin B12-dependent enzyme methionine synthase catalyzes the transfer of the methyl group from N-5-methyl-tetrahydrofolate to Hcy to form methionine. Vitamin B12 deficiency can lead to homocysteine remethylation defects, resulting in the accumulation of Hcy. Post-methionine-load hyperhomocysteinemia may be due to a heterozygous cystathionine β-synthase defect or B6 deficiency^17^. Other factors that control plasma Hcy levels include impaired renal function, high plasma creatinine, smoking, coffee consumption, alcoholism, and certain drugs^17^. However, previous studies have not investigated these factors in males and females separately. As a result of this lack, it is necessary to separately investigate the factors influencing Hcy levels in males and females to see if and how they differ.

## Results

### Characteristics of the participants

**Figure 1:**
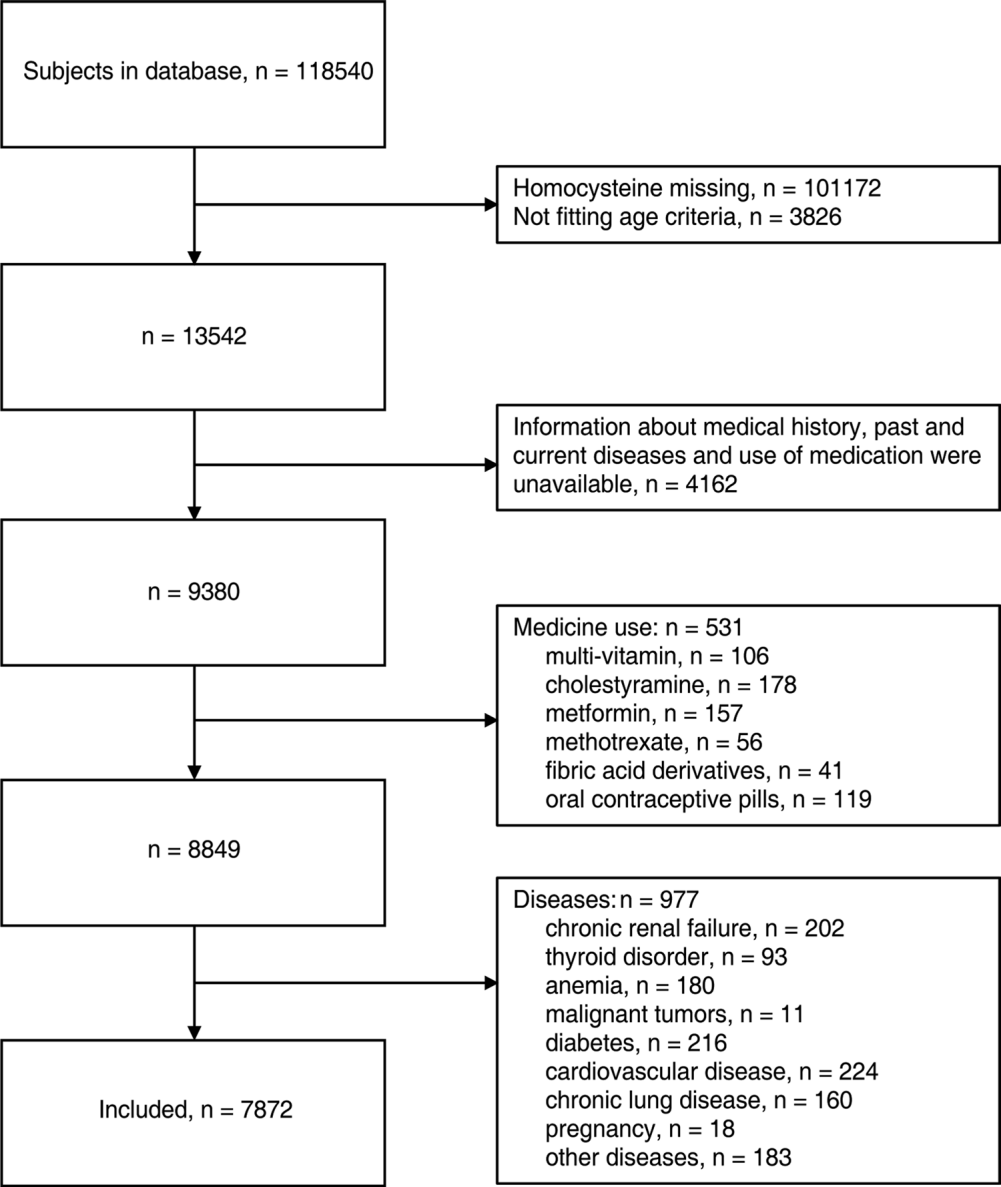
Flow diagram describing the selection strategy.

A total of 7872 individuals from the Health Management Center of Tongji Hospital participated in this study. Their mean age was 49.8 ± 11.6 years; 4999 (63.3%) were males, 2873 (36.7%) were females. The participants were divided into two groups, male and female, according to gender. The baseline characteristics of the participants in each group are presented in Table 1. The median of the plasma Hcy was 12.5 umol/L in males and 9.1 umol/L in females. Geometric mean Hcy concentrations were 13.5 umol/L in males and 9.7 umol/L in females. Independent Student’s *t*-test and Manne-Whitney U test showed significant differences between males and females in most anthropometric and laboratory indices. Of these, body mass index (BMI), systolic blood pressure (SBP), diastolic blood pressure (DBP), free triiodothyronine (fT3) level, Cystatin C, creatinine, uric acid, blood urea nitrogen (BUN) and Hcy levels were higher in males, whereas estimated glomerular filtration rate (eGFR) and thyroid stimulating hormone were higher in females (Table 1) (Fig. 1).

**Table 1:** Characteristics of the study population.

Characteristics	Men (N = 4993)	Women (N = 2879)	*P* value
Age (years)	48.54 ± 10.84	52.28 ± 12.5	< 0.0001
BMI (kg/m^2^)	25.38 ± 3.11	23.44 ± 3.17	< 0.0001
SBP (mmHg)	128.71 ± 18.03	125.75 ± 21.24	< 0.0001
DBP (mmHg)	80.07 ± 12.48	73.95 ± 12.34	< 0.0001
Homocysteine (umol/L)^a^	12.5 (6.0)	9.1 (2.9)	< 0.0001
Cystatin C (mg/L)	0.94 ± 0.17	0.83 ± 0.16	< 0.0001
Creatinine (umol/L)	83.88 ± 14.13	61.50 ± 11.51	< 0.0001
UA (umol/L)	390.10 ± 84.44	279.14 ± 67.24	< 0.0001
BUN (mmol/L)	5.23 ± 1.29	4.8 ± 1.32	< 0.0001
eGFR (ml/min/1.73 m^2^)	96.92 ± 18.81	111.96 ± 24.79	< 0.0001
fT3 (pg/mL)	2.97 ± 0.77	2.78 ± 0.75	< 0.0001
fT4 (ng/dL)	1.02 ± 0.16	1.02 ± 0.15	0.163
TSH (uIU/mL)	2 ± 2.79	2.48 ± 3.77	< 0.0001
TP (g/L)	74.82 ± 3.97	75.44 ± 4.2	< 0.0001
ALB (g/L)	45.72 ± 2.53	44.57 ± 2.46	< 0.0001
Glubulin (g/L)	29.1 ± 3.54	30.86 ± 3.76	< 0.0001
K (mmol/L)	4.37 ± 0.31	4.27 ± 0.3	< 0.0001
Ca (mmol/L)	2.81 ± 0.91	2.81 ± 0.88	0.831
Smoker^b^	2307 (46.20%)	99 (3.44%)	< 0.0001
Drinker^b^	2761 (55.30%)	492 (17.09%)	< 0.0001

Values are expressed as mean ± SD unless otherwise specified.

BMI, body mass index; SBP, systolic blood pressure; DBP, diastolic blood pressure; UA, uric acid; BUN, blood urea nitrogen; eGFR, estimated glomerular filtration rate; TSH, thyroid stimulating hormone; fT3, free triiodothyronine; fT4, free thyroxine; TP, total protein; ALB, albumin; K, serum potassium; Ca, serum calcium.

^a^Values are expressed as median (interquartile range) due to significant skewness.

^b^Values are expressed as n (%). *P* values were based on independent two sample *t* test. ^a^Manne-Whitney U test. ^b^Chi-square test.

### Age-related change of homocysteine concentrations in males and females

**Figure 2:**
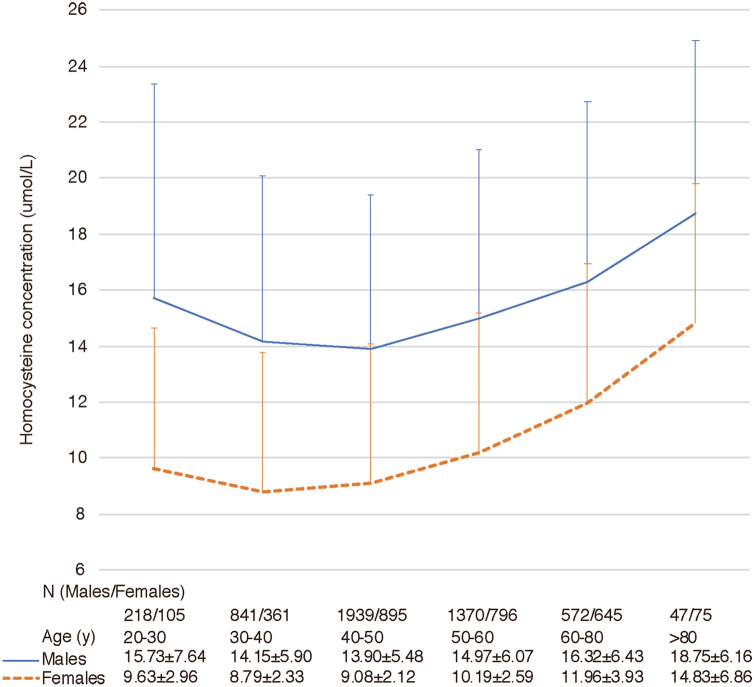
The age-related change of homocysteine concentration. Data are expressed as means ± SD.

Gender -and age-related changes in Hcy concentration are shown in Fig. 2. The results showed that the human serum Hcy concentration first decreases and then increases during the lifetime. It is the lowest between 30 and 50 years of age and increases significantly after 50 years of age. The difference between genders in the serum Hcy concentration level in each age range was significant (*P* < 0.0001). Moreover, the mean Hcy levels in adult males less than 30 years of age and greater than 60 years were higher than the upper limit of normal (15 umol/L).

### Hyperhomocysteinemia in males and females

**Figure 3:**
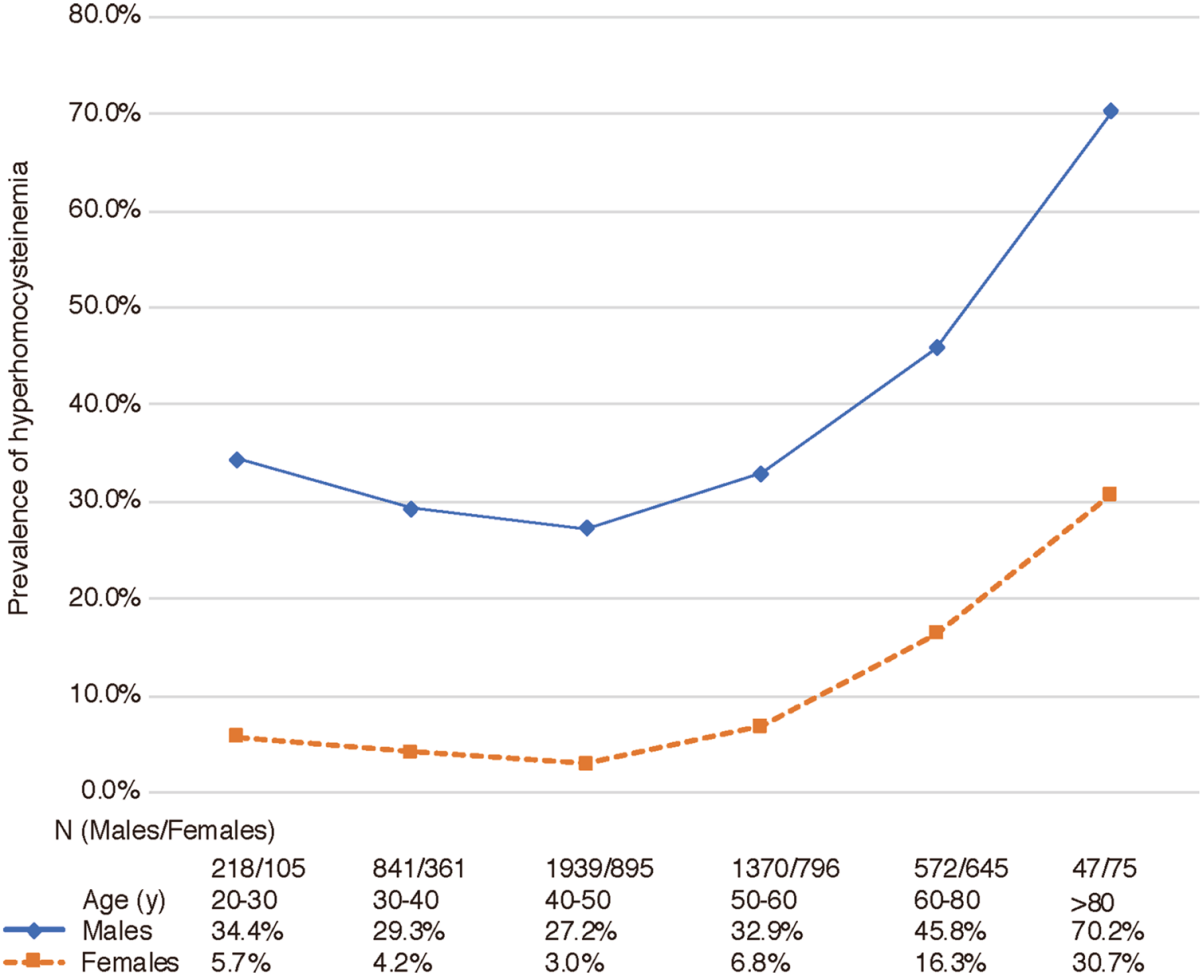
The prevalence of hyperhomocysteinemia in males and females. Hyperhomocysteinemia was defined as homocysteine serum levels > 15 umol/L.

The proportion of hyperhomocysteinemia in males was significantly higher than in females in each age range (all *P* < 0.0001), as shown in Fig. 3. The prevalence of hyperhomocysteinemia in males remained at a rather high level in all age ranges; the lowest value was 27.2% at 40 to 50 years of age. In females, the prevalence of hyperhomocysteinemia remained at a low level in all age ranges; the highest value was 30.7%, at over 80 years of age. According to logistic regression analyses, males have a higher risk of hyperhomocysteinemia than females in all age ranges (Table 2). The overall odds ratio (OR) value was 4.33 (95% CI: 2.71–6.83; *P* < 0.0001) and 2.23 (95% CI: 1.12–4.10; *P* < 0.0001) before and after adjustment for age, BMI, smoking status, drinking, eGFR and creatinine, respectively. Males between 18 and 35 years of age had the highest risk of hyperhomocysteinemia compared with females in the same age range, with an adjusted OR value of 4.14 (95% CI: 2.16–8.21; *P* < 0.0001).

**Table 2:** Logistic regression analyses of the risk of hyperhomocysteinemia.

Hyperhomocysteinemia	18 years ≤ Age < 35 years (N = 740)	35 years ≤ Age < 55 years (N = 4811)	55 years ≤ Age (N = 1892)	All subjects (N = 7449)
Unadjusted	5.61(2.86–9.16)	4.56(2.14–7.85)	3.16(1.54–5.05)	4.33(2.71–6.83)
Adjusted	4.14(2.16–8.21)	2.41(1.51–5.76)	1.89(1.05–3.10)	2.23(1.12–4.10)

Hyperhomocysteinemia: homocysteine serum levels > 15 mmol/L. Adjusted model: adjusted for age, body mass index, smoking status, drinking, eGFR and creatinine.

Odds ratios for men in comparison to women, with adjustments and 95% confidence.

### Hyperhomocysteinemia at different ages

The prevalence of hyperhomocysteinemia was lowest at 30 to 50 years of age, both in males (29.3–27.2%) and in females (4.2–3.0%) and began to rise after 50 years of age, reaching 70.2% and 30.7%, respectively, in males and females more than 80 years of age (Fig. 3). The association between age and hyperhomocysteinemia was examined using logistic regression (Table 3). The odds ratio of hyperhomocysteinemia for males aged 55 years and older compared to those aged between 35 and 55 years was 1.825 (95% CI 1.548–2.152), while there was no significant hyperhomocysteinemia risk in males aged between 18 and 35 years, compared to those aged between 35 and 55 years. For females aged between 18 and 35 years and those aged 55 years and older there was a high risk of hyperhomocysteinemia compared to females aged between 35 and 55 years, with an OR of 1.803 (1.267–2.566), 2.43 (1.223–4.828) and 3.430 (2.723–4.128), respectively.

**Table 3:** Logistic regression analyses of the risk of hyperhomocysteinemia between ages.

	Male		Female	
	OR (95% CI)	*P*	OR (95% CI)	*P*
35 years ≤ Age < 55 years	Reference		Reference	
18 years ≤ Age < 35 years	1.149(0.919–1.437)	0.224	1.803(1.267–2.566)	0.011
55 years ≤ Age	1.825(1.548–2.152)	< 0.0001	3.430(2.723–4.128)	0.001

Hyperhomocysteinemia: homocysteine serum levels > 15 mmol/L. Adjusted for age, body mass index, smoking status, alcohol drinking, eGFR and creatinine.

### Independent factors affecting Hcy concentration in males and females

Independent factors associated with an increased Hcy concentration in males were smoking status (current smokers versus ex-smokers: β: 0.112, *P* = 0.003), decreased eGFR (*β* =  − 0.192, *P* < 0.0001), BUN (*β* =  − 0.14, *P* = 0.003), DBP(*β* =  − 0.113, *P* = 0.01), fT3 (*β* =  − 0.091, *P* = 0.038), serum potassium (K) (*β* =  − 0.107, *P* = 0.015) and increased cystatin C (*β* = 0.173, *P* = 0.002). In females, independent factors associated with an increased Hcy concentration were increased cystatin C (*β* = 0.319, *P* < 0.0001), albumin (*β* = 0.227, *P* < 0.0001), free thyroxine (fT4) (*β* = 0.179, *P* = 0.004), age (*β* = 0.148, *P* = 0.037), and decreased fT3 (*β* =  − 0.217, *P* = 0.001) and K (*β* =  − 0.153, *P* = 0.008) (Table 4). Adjusted R^2^ was used to test the goodness of fit of the regression model. The R^2^ for the regression model of males and females were 0.204 and 0.250, respectively.

**Table 4:** Multivariate analysis of factors affecting Hcy concentration in male and female.

	Male		Female	
	β	*P*	β	*P*
eGFR	− 0.192	< 0.0001	–	–
Cystatin C	0.173	0.002	0.319	< 0.0001
BUN	− 0.14	0.003	–	–
Albumin	–	–	0.227	< 0.0001
DBP	− 0.113	0.01	–	–
Smoker (vs. ex-smoker)	0.112	0.003	–	–
fT3	− 0.091	0.038	− 0.217	0.001
fT4	–	–	0.179	0.004
K	− 0.107	0.015	− 0.153	0.008
Age	–	–	0.148	0.037

Multivariable analysis included smoking status, drinking, age, body mass index, systolic blood pressure, DBP, blood urea nitrogen, creatinine, eGFR, cystatin C, thyroid stimulating hormone, fT3, fT4, serum potassium, total protein, albumin. Values of β are standardized regression coefficients. A smoker in this study means a current smoker; ex-smoker includes never smoker and former smoker.

eGFR, Estimated glomerular filtration rate; BUN, blood urea nitrogen; DBP, diastolic blood pressure; fT3, free triiodothyronine; fT4, free thyroxine; K, serum potassium.

## Discussion

This study was conducted to investigate the distribution of plasma Hcy concentrations in males and females in China and its change with age and to explore independent factors affecting the Hcy concentration. A total of 7872 individuals from among a total of 116,940 people participated in this study. The proportion of males participating was higher (63% of the cohort) than in the general Chinese population, which is about 51.1% according to the National Bureau of Statistics (https://www.stats.gov.cn). This may be because some of the people who visited the hospital for a routine health check-up were referred by their employers and men have higher employment rates than women.

The results of the data analysis showed that the plasma Hcy levels first decreased and then increased, in both males and females, being lowest at 30–50 years of age and increasing significantly after 50 years of age. The trend differs from studies of the white and black populations in the United States, which showed that Hcy concentrations increase with age throughout adulthood^15^. The mean Hcy concentration levels also showed racial and ethnic differences. The geometric mean plasma tHcy level (13.5 umol/L for males and 9.7 umol/L for females) for our subjects tended to be higher compared with the geometric mean values in the U. S. NHANES report^15^ (9.6 and 7.9 umol/L in non-Hispanic white males and females, respectively, 9.8 and 8.2 umol/L in non-Hispanic black males and females, respectively, and 9.4 and 7.4 mmol/L in Mexican American males and females, respectively), geometric means in the Hordaland Homocysteine Study for Norway^5^ (10.8 umol/L for men and 9.1 umol/L for women), and geometric means reported in Korean adults^18^ (11.18 umol/L for men and 9.20 umol/L for women). The data indicate that the reference range of Hcy concentration in Western countries may not be applicable to Chinese people.

Based on the analysis of Hcy levels in men and women in this study, the results showed definite differences by gender. In comparison with females, Hcy levels in males were significantly higher at each age range, and the trend did not abate with age. In addition, the percentage of males with hyperhomocysteinemia was also higher than that of females at all ages. Previous studies have reported possible associations between various endogenous sex hormones and Hcy levels. Studies have shown that males have higher concentrations of Hcy than females, but the difference diminishes after menopause^19^. However, our study shows that gender-related differences persist over the lifespan, eliminating the beneficial effects of estrogen. This is consistent with the results reported by Cohen et al.^14^, which indicates that these gender-related differences persisted in a subgroup analysis of the subjects above the age of 55 years. In another study on sex hormones and the Hcy level in older males, the results did not support a direct role of circulating sex hormone levels in regulating the fasting Hcy concentration in middle-aged and older males^20^. Our collinearity analysis showed independent effects of age and gender in the relationship with Hcy.

It is well known that vitamin B12 deficiency is a major contributor to elevated Hcy levels. Deficiency of vitamin B12, an important methyl-cobalamin synthesis enzyme, can lead to impaired Hcy re-methylation and Hcy accumulation^17^. Additionally, vitamin B12 deficiency was found to be significantly associated with gender, and the mean values of serum vitamin B12 concentration for men are lower than those for women^21,22^. The above suggest that vitamin B12 may explain the relationship between gender and Hcy concentration. The values of serum vitamin B12 in this study were not recorded, which limits the current study. However, according to one previous study, men still had a higher OR (3.44; 95% CI, 2.89–4.09) for hyperhomocysteinemia compared to women, after adjusting for vitamin B12 and some other related confounders (age, smoking status, kidney function and folate). Even if vitamin B12 plays a role in the relationship between gender and Hcy concentration, it seems that the findings of the current study cannot be explained solely by vitamin B12.

**Figure 4:**
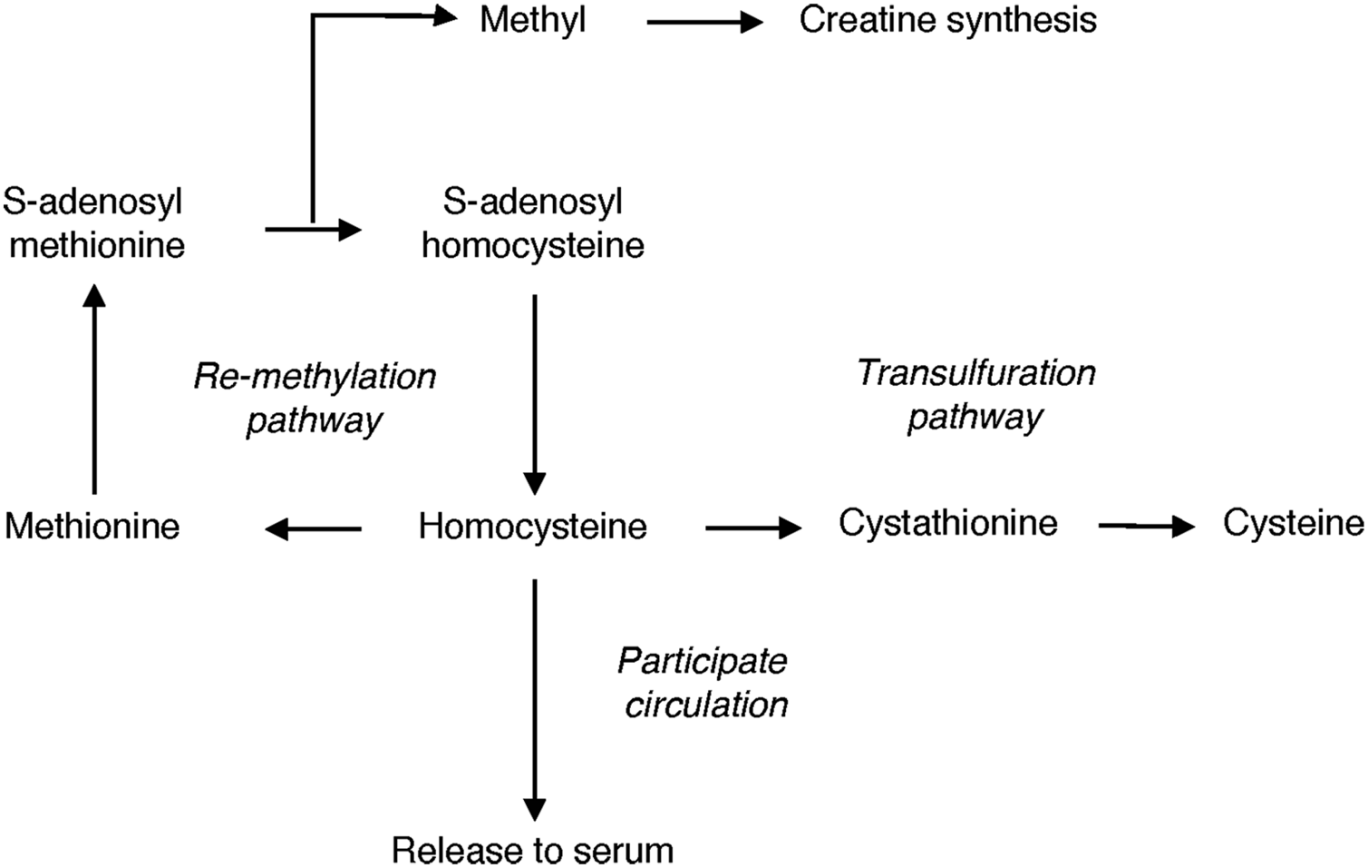
Pathways of homocysteine metabolism.

These highlight that gender per se is a possible unique independent factor in Hcy concentration and perhaps the difference in Hcy concentration between males and females can be explained by gender-related differences in Hcy metabolism. We noticed that males need to produce creatine more often than females because they have more muscle mass^23^. Part of the methyl donor needed for creatine synthesis comes from s-adenosyl methionine conversion to s-adenosyl homocysteine. S-adenosyl homocysteine is the precursor of Hcy^24^, as shown in Fig. 4. Contrarily, studies have shown that females have a greater Hcy flux through the transsulfuration pathway, which lowers the Hcy concentration^25^.

According to the data analysis, age also has a significant effect on serum Hcy concentration. After 50 years of age, the Hcy concentration is significantly higher than in other age groups. We speculate there are two reasons for this: first, decreased liver and renal function induce decreased Hcy metabolism in the elderly, resulting in increased serum Hcy concentration; second, the digestive and absorption dysfunction of the elderly lead to vitamin B12 and folate deficiencies, which affects Hcy metabolism. This suggests that clinicians should pay special attention to elderly patients, especially those with cardiovascular and cerebrovascular diseases, as an elevated plasma Hcy level has been reported to be associated with major components of the cardiovascular risk profile^5,26^ and endothelial dysfunction^27^. However, it is worth noting that in the multivariate regression analysis of males and females, age is an independent risk factor for homocysteine only in females. In males, age is significant only before the confounders were adjusted, which seems inconsistent with the relationship between age and Hcy concentration shown in the figures.

Independent factors affecting the Hcy concentration in males and females were explored. The results showed that these factors were different in males compared to females. In males, factors associated with Hcy concentration were eGFR, BUN, cystatin C, DBP, smoking status, fT3 and K, while in females, factors associated with Hcy concentration were, cystatin C, albumin, fT4, fT3, age, and K. Additionally, a previous study found that high Hcy levels were negatively associated with cardiovascular health in females, but not in males^28^. This indicates that not only are the factors affecting Hcy in males and females different, but also, the effects on the body are different, which is very interesting and worth further study.

A previous study showed a negative linear association between the plasma Hcy level and GFR^29^. Hyperhomocysteinemia occurs when the GFR is about 60 mL/min, and the possibility of hyperhomocysteinemia in end stage renal disease is 85–100%^30^. Proposed mechanisms for the relationship between renal function and Hcy include reduced renal elimination of Hcy and damaged non-renal disposal^31^. Damaged non-renal disposal includes the inhibition of crucial enzymes in Hcy metabolism^31^ and abnormal folate metabolism when in renal failure^30^. There was one notable contradictory result in that BUN was negatively correlated with Hcy, which should be positively correlated according to the above theory. Urea is the main end product of protein metabolism and Hcy is a methionine metabolism intermediate, which mainly binds to albumin by the disulfide bond. We hypothesized that BUN levels increase when the body is in a high catabolic state, and Hcy levels decrease as a result of decreased protein levels. This assumption, however, was not confirmed.

fT3 was negatively correlated with Hcy in both men and women. Increased fT4 was associated with increased Hcy in women. Studies on thyroid function and Hcy have shown increased Hcy levels in patients with hypothyroidism and subclinical hypothyroidism^32,33^, and low Hcy levels in patients with hyperthyroidism^34^. A study^35^ reported negative correlations between Hcy and TSH and fT3, and a positive correlation between fT4 and Hcy. However, only the data exists and no theory was proposed. Whether there is a causal relationship between Hcy concentration and thyroid function needs further investigation. It is interesting that the average fT3 level of men is higher than that of women^36^, but the higher fT3 levels do not help men have a lower Hcy level than women. It may be that other factors that increase Hcy in men mask this effect.

In addition, multiple linear regression analysis showed that the Hcy concentration was positively correlated with albumin levels in females. Hcy is an intermediate product of methionine metabolism. In vivo, it is mainly bound to albumin by the disulfide bond, and very little Hcy is in the free state. Table 2 shows that the average albumin level in females is lower than in males. Whether the lower Hcy level is related to the low albumin level remains to be confirmed. Furthermore, according to the molecular studies on Hcy toxicity, protein N-homocysteinylation is one of the important pathogenic and toxic mechanisms of hyperhomocysteinemia^37^. It has been proved that Hcy and its derivatives can modify protein targets including albumin, hemoglobin, low density lipoprotein, cytochrome C, C-reactive protein, etc.^38–41^. Protein N-homocysteinylation provided another perspective for us to analyze the gender difference in hyperhomocysteinemia.

Serum potassium was negatively correlated with Hcy in both men and women. As far as we know, there is no research showing that serum potassium has a biological effect on Hcy concentrations. But when potassium was added to the multiple regression model, the regression coefficients of the other variables in the model changed greatly and the goodness of fit of the model improved. Even with a sample of such magnitude, significant associations might be random, particularly for associations which carry no biological plausibility. Considering that the inclusion of potassium to the model only affects the size of the regression coefficient of other variables, but not its significance, we displayed the results that contained potassium just in case there are biological processes associated with potassium and Hcy that we don’t know yet.

This study is the first statistical analysis of the Hcy concentration over the lifetime in males and females, separately, and the first investigation of the factors associated with the Hcy concentration. We found significant differences in Hcy level between males and females, which differences did not diminish in old age. At the same time, the relationship of thyroid function and albumin with Hcy was confirmed for the first time, providing abundant reliable data for further understanding Hcy and clues and ideas for further exploration of the effects of Hcy on the body.

Our study has some limitations that deserve mentioning. First, we were unable to infer causality because this was a retrospective cross-sectional study. However, the examined association implies causation. Second, important parameters (vitamin B12 and folate levels, sex hormones, etc.) should be framed as residual confounding to avoid a false positive (Type I) error. However, we can only discuss confounding factors qualitatively rather than quantify their effects because they were not recorded during the health checks. The third limitation concerns the external validity of the study. Because Hcy tests are only given to individuals or groups that have purchased more expensive medical checkup packages, the subjects in this study may not be a good representation of different socioeconomic classes. However, although the external validity of the study is limited, it is not expected to influence the examined associations. Last, the relationship between serum potassium and Hcy concentration may be only at the analytical level.

## Conclusion

In summary, this population-based, cross-sectional study showed that Hcy concentrations first decreased and then increased, being minimum at 30–50 years of age and significantly increasing after 50 years of age. Hcy levels in males were significantly higher than in females in each age range. The trend did not abate with age; instead, the levels for males remained above normal. This may be a contributing factor to gender differences in atherosclerosis and coronary artery disease.

## Methods

### Data source

This retrospective study used data from the Health Management Centre of Tongji Hospital, Huazhong University of Science and Technology, Wuhan, Hubei, China. Tongji Hospital, which was founded in 1900, is the largest tertiary medical center in central China. The Health Management Centre of Tongji Hospital, with three branches, covers the entire Wuhan region, including the Jiangan District, Jianghan District, Qiaokou District, Hanyang District, Wuchang District, Chingshan District, Hongshan District, Caidian District, Jiangxia District, Huangpi District, Xinzhou District, Dongxihu District and Hannan District. Nowadays, routine health examinations have become very common in China, especially in big cities like Wuhan, as the central government is encouraging people to get regular medical checkups. The Tongji Hospital-Health Management Center provides routine medical examination service for individuals and groups, and has a database (TJ-HMCD) containing the electronic health records of persons of most occupations and retired persons living in residential communities of Wuhan. Therefore, the sample is comparatively representative and comprehensive. There are four optional medical checkup packages provided by the Health Management Centre: basic package, standard package A, standard package B and standard package C. Individuals or groups who buy packages B or C will receive more comprehensive health checks, in which homocysteine is tested as a risk marker for cardiovascular and cerebrovascular disease.

Data for this study were obtained from Database 2018 (TJ-HMCD2018) covering persons who received health screening within the time frame of January 1, 2018 to December 31, 2018. The HMCD2018 includes data of 118,540 persons and represents about 1.07% of the total population of Wuhan. There are no significant differences in age, gender, or healthcare costs between the TJ-HMCD2018 and the whole database (TJ-HMCD). The study protocol was approved by the Ethics Committee of Tongji Hospital, Tongji Medical College, Huazhong University of Science and Technology (TJ-IRB20191012). All research was performed in accordance with relevant guidelines and regulations. To protect patient privacy, all personal identification information was deleted before being released for research. Considering that the data for this study does not include personal identifying information (e.g., name, address, and identification number), the Ethics Committee of Tongji Hospital, Tongji Medical College, Huazhong University of Science and Technology waived the requirement for written informed consent for this de-identified study.

### Study sample

Subjects were recruited who were over 18 years of age and who completed the laboratory measurements, including serum Hcy. In addition, information about demography, health-related habits, medical history, past and current diseases and use of medication were available. Exclusion criteria were: being treated for hyperhomocysteinemia or current users of drugs known to influence Hcy concentration, including multi-vitamin (vitamin B6, B12, folate, niacin, or any other vitamin), cholestyramine, metformin, methotrexate, fibric acid derivatives, and oral contraceptive pills. In addition, subjects with self-reported or health examination suggesting chronic renal failure, thyroid disorder, anemia, malignant tumors, diabetes, CVD, chronic lung disease, pregnancy, or other diseases that might affect Hcy concentration or metabolism were excluded. A flow diagram describing the detailed information about the selection strategy is shown in Fig. 1.

### Measurement of homocysteine

Venous blood samples were collected with anticoagulant tubes after an overnight 12-h fast. The samples were centrifuged within an hour (30 min, RT, at 3000RPM; Centrifuge, DT5-4B, Beili Co., Beijing, China) and tested within two hours. The serum Hcy was measured with an assay kit (DiaSys Diagnostic Systems, Shanghai, China) based on an enzymatic cycling method. The reagent detection range was 1–50 umol/L. When the measured value of the sample exceeded the upper limit, it was diluted and remeasured. The sensitivity was 1 umol/L. The intra-assay and inter-assay coefficients of variation (CV%) were 2.2–4.8% and 3.2–5.5%, respectively. The measurement was performed on Roche COBAS c502 (Swiss). Quality control was conducted first every day. Specimen testing was conducted only after qualified quality control. Every month, statistical analysis of the quality control chart was conducted to ensure the stability of the quality control results. Every year, the laboratory participates in external quality assessment (EQA) conducted by the inspection center of the Ministry of Health to ensure the accuracy of the test results. The normal range of Hcy concentrations is between 5 and 15 umol/L and above 15 umol/L is considered to be hyperhomocysteinemia^15^.

### Measurement of other variables

On the day of examination at the screening center, the visitors were interviewed by registered nurses to acquire the demographic information (e.g., age, gender), health-related habits (e.g., smoking status, alcohol drinking status) and physiologic status (e.g., pregnancy, fasting time) by referring to a standard questionnaire. Medical history, past and current diseases and use of medication were inquired and recorded by trained nurses using another questionnaire. Smoking status was classified as never smoker, current smoker or former smoker. A smoker in this study means a current smoker; ex-smoker includes never smoker and former smoker. Drinker and non-drinker were determined based on whether a person had consumed at least 12 drinks of any type of alcoholic beverage in the previous year. The height and body mass were measured by a stadiometer after fasting, without coat and shoes. BMI was calculated by dividing weight (kg) by height squared (m^2^). Blood pressure was measured using an electronic sphygmomanometer (HBP-9020; Omron, Dalian, China) on the right arm in a seated position after 10 min of rest. Venous blood samples for routine biochemical test (e.g., fasting blood glucose, total cholesterol, triglyceride, high-density lipoprotein cholesterol, low-density lipoprotein cholesterol, urea nitrogen, creatinine, and uric acid) were performed at the accredited hospital laboratories with standard methods on an automatic biochemistry analyzer (Roche Cobas 8000 modular analyzer). eGFR was calculated based on the Modification of Diet in Renal Diseases study equation^42^.

### Statistical analysis

Descriptive statistics are expressed as mean ± standard deviation (SD) for continuous variables, or as median and interquartile range if significant deviation from normal distribution. Categorical variables were presented as frequency and percentages. The differences in population characteristics between men and women were compared using unpaired Student’s *t* test, nonparametric Mann–Whitney U-test for continuous variables as appropriate, and Chi-square test or Fisher's exact for categorical variables. The distributions of serum Hcy in both men and women were skewed to the right, therefore, values were natural-log-transformed to promote normality and assessed by a Kolmogorov–Smirnov test. The means and SD of the Hcy concentration in different age groups were calculated according to the following formulas^43^. $\bar{{\text{X}}} = {\text{e}}^{{{\upmu } + \frac{1}{2}{\upsigma }^{2} }}$, $\mathrm{SD }= {\text{e}}^{{{\upmu } + \frac{1}{2}{\upsigma }^{2} }} \sqrt {{\text{e}}^{{{\upsigma }^{2} }}  - 1}$ (μ and σ are location and scale parameters for the normally distributed logarithm ln X, calculated by natural-log-transformed Hcy). Odds ratios (ORs) and 95% confidence intervals (CIs) obtained from logistic regression models were used to predict hyperhomocysteinemia risk in relation to gender and age, with and without correction. Multiple linear regression was performed to identify the independent factors that affect the Hcy concentration for males and females. Candidate variables with a *P* value < 0.2 on univariate analysis or considered clinically relevant were included in multivariable model; Confounding factors that cannot be determined to enter the model were put into the model to see if they cause significant changes in the model. The final models were determined by a stepwise variable selection approach. Standardized regression coefficients were used and calculated as the non-normalized regression coefficients times the SD of the independent variable and then divided by the SD of the dependent variable. Tolerance and variance inflation factor (VIF) were used to evaluate the interaction between age and gender in the relationship with Hcy. If the tolerance < 0.1 or VIF > 10, it means collinearity exists. All statistical tests were two-sided and *P* < 0.05 was considered to indicate statistical significance. Data management and analysis were performed using IBM SPSS software version 23.0 (IBM Inc., Armonk, NY, USA).

## Data Availability

The data that support the findings of this study are available from the corresponding author upon reasonable request and with permission of the Health Management Centre of Tongji Hospital.
